# Impact of Economic Policy Uncertainty on Carbon Emissions: Evidence from 137 Multinational Countries

**DOI:** 10.3390/ijerph19010004

**Published:** 2021-12-21

**Authors:** Hai-Jie Wang, Yong Geng, Xi-Qiang Xia, Quan-Jing Wang

**Affiliations:** 1 School of Business, Zhengzhou University, 100 Kexue Road, Gaoxin District, Zhengzhou 450001, China; whj@zzu.edu.cn (H.-J.W.); xqxia@zzu.edu.cn (X.-Q.X.); 2 School of Environment and Science Engineering, Shanghai Jiaotong University, 800 Dongchuan Road, Minhang District, Shanghai 200240, China; ygeng@sjtu.edu.cn

**Keywords:** economic policy uncertainty, carbon emissions, GMM, multinational research

## Abstract

With growing economic policy uncertainty (EPU) and the importance of protecting the natural environment worldwide, the relationship between EPU and carbon emissions should be investigated further. However, conclusions in the existing literature on the relationship between EPU and carbon emission are inconclusive. This paper aims to examine the influence of EPU on carbon emissions according to the Stochastic Impacts by Regression on Population, Affluence and Technology (STIRPAT) model. To investigate such essential issues, we conduct GMM estimations by utilizing cross-country data covering 137 countries during the period 1970–2018, obtained from World Bank and OECD statistics. Our empirical estimations support that EPU would bring about more carbon emissions, while we conduct empirical analysis by changing the system of measurement, employing alternative estimation and constructing new samples. Our study provides substantial policy implications for government participation in international treaties on environmental protection to mitigate environmental degradation.

## 1. Introduction

Nowadays, due to the outbreak of the COVID-19 pandemic and growing tension concerning the international environment, economic policy uncertainty (EPU) is growing rapidly worldwide (Baker et al., 2020; Jordà et al., 2020; Bakas and Triantafyllou, 2020) [1,2,3]. EPU can not only change economic activities or the attention of policymakers (Balcilar et al., 2016; Degiannakis et al., 2018; Hailemariam et al., 2019; Phan et al., 2021) [4,5,6,7], but also change a firm’s behaviors or decisions in the direction of environmental protection (Guidolin and La Ferrara, 2010; Kang et al., 2017; Olanipekun et al., 2019; Akron et al., 2020) [8,9,10,11], since economic activities and the manner of production contribute to air pollution (Salahuddin et al., 2018; Shahbaz et al., 2019) [12,13]. With growing social problems such as human diseases, extreme climate events, and natural disasters caused by serious air pollution (Kompas et al., 2018; Shahbaz et al., 2019; Coskuner et al., 2020) [13,14,15], it is generally accepted globally that to reduce air pollution is essential for human survival and for national sustainable development (Hambira et al., 2020; Huo et al., 2020) [16,17]. Thus, it is necessary to query whether increasing EPU can affect air pollution (Jiang et al., 2019; Yu et al., 2021; Adams et al., 2020) [18,19,20].

In fact, a small number of studies have begun to focus on the relationship between EPU and air pollution, especially after the research of Jiang et al., (2019) [18] who declared that EPU can affect direct government policy, which may promote or hinder carbon emissions. Later, scholars began to query the effect of EPU on CO_2_ emissions. However, there are some gaps in the existing studies. Specifically, the conclusions are controversial (Gill et al., 2019; Shabir et al., 2021) [21,22], and while much of the literature has pointed out that EPU brings more environmentally adverse effects (Yu et al., 2021, Adedoyin and Zakari, 2021) [19,23], a small number of scholars have argued that EPU does some good in reducing CO_2_ emissions (Chen et al., 2021; Doğan and Güler, 2021) [24,25], and other researchers argue that there is no influence of EPU on CO_2_ emissions (Abbasi and Adedoyin, 2021; Liu and Zhang, 2021) [26,27]. Additionally, most studies have utilized the EPU index given by Baker et al., (2016) [28] to measure EPU, but this index has some limitations in that it cannot reflect uncertainty related to political events and it is not calculated from a single base for different countries worldwide, which may bring about biased results (Adams et al., 2020; Atsu and Adams, 2021) [20,29]. Besides, empirical investigations of the impact of EPU on CO_2_ emissions are utilizing simple data for one or a few countries (Pirgaip and Dinçergök, 2020; Adedoyin and Zakari, 2021; Sohail et al., 2021) [23,30,31]. Aside from these, some studies have conducted an empirical investigation into this issue by employing the Stochastic Impacts by Regression on Population, Affluence and Technology (STIRPAT) model, which is generally utilized to conduct empirical investigation for issues such as CO_2_ emissions (Amin and Dogan, 2021; Anser et al., 2021; Yu et al., 2021) [19,32,33].

This scenario naturally motivates us to raise an interesting issue: what effect does EPU exert on air pollution? To answer this, we conducted an empirical investigation by employing multinational panel data for 137 countries during the period 1970–2018 via GMM estimation according to the STIRPAT model. We measured EPU by the variable World Uncertainty Index provided by Ahir et al., (2018) [34], which can help us to comprehensively examine the role of EPU in air pollution. Compared to the previous literature, the potential contributions of this work are as follows. Firstly, we carry out empirical estimation for the impact of EPU on air pollution based on multinational panel data for 137 countries via the world uncertainty index for EPU provided by Ahir et al., (2018) [34], which can offer further common findings on EPU’s influence on air pollution (Chen et al., 2021) [24]. Secondly, only a few studies have utilized the STIRPAT model, which is generally utilized in order to investigate the influence of EPU on carbon emissions, and in this study we attempted to examine the impact of EPU on carbon emissions by conducting static and dynamic estimations using the STIRPAT model.

The rest of this paper is structured in line with previous empirical studies. Specifically, Section 2 is mainly concerned with the variable data and estimation method. Section 3 offers the main results and discussion. Section 4 gives a brief summary of our empirical results and proposes some suggestions to policymakers.

## 2. Materials and Methods

### 2.1. Variables

Air pollution (CO_2_): In line with Shao et al., (2011) [35], CO_2_ emission is the most critical among environmental pollutants and is generally accepted as an indicator of environmental impact or climate change. Due to the well-established database on CO_2_ emission, we also measure CCP by CO_2_ emissions (denoted by CO_2_) (the unit of CO_2_ is one million tons).

Economic policy uncertainty (Uncertainty): variables for EPU such as the EPU index, world trade uncertainty index (WTUI) and world uncertainty index (WUI) were generally utilized in previous studies investigating the issues of EPU (Pirgaip and Dinçergök, 2020; Adams et al., 2020; Qi et al., 2021) [20,30,36]. However, the data for EPU only covers 28 countries, while the data for WTUI and WUI was constructed for 143 countries from 1996 onwards. Besides, compared to the EPU index, the WUI index can capture the uncertainty of political events and is built according to a common base for all countries (Atsu and Adams, 2021) [29]. To come to conclusions on the role of EPU in carbon emissions worldwide, we utilize the WUI data to measure the EPU in line with Adams et al. (2020) [20]. Specifically, Ahir et al. (2018) [34] construct quarterly indices of EPU by using frequency counts of “uncertainty” (and its variants) in the quarterly Economist Intelligence Unit (EIU) country reports, which are scaled by the total number of words in each report and multiplied by 1000 for worldwide comparability. The EPI data is year-frequency, and takes the mean value of quarterly WUI to measure EPU annually (Atsu and Adams, 2021) [29], denoted by Uncertainty. A higher value of Uncertainty usually stands for higher EPU.

According to previous articles focusing on carbon emissions and the extended STIRPAT model (Wen et al., 2016; Wang et al., 2021b; Yang et al., 2021) [37,38,39], we include the following variables in our model:

Economic performance (GDP): As suggested in the traditional STIRPAT model, Affluence is usually measured by national real income or GDP per capital, so in this study we also measure Affluence by the national real GDP per capita, which is constant in 2011 US dollars and denoted by GDP.

Population (POP): In the STIRPAT model, Population is often captured by the total number of people; to control the influence of population on carbon emissions, we also utilize this indicator to measure population, denoted by POP (Verma et al., 2021) [40].

Green technologies (GI): In the STIRPAT model, T often stands for technologies which can produce goods in an environmentally-friendly manner or that increase energy efficiency which can reduce carbon emissions. In this study, we capture T by green technologies, measured by the total amount of patent applications in environmental management, denoted by GI.

Density of people (Density): Aside from the total population, we also measure population by density, as higher density may bring about more demand in terms of deforestation in order to offer more space for human living. As provided by Bottero et al., 2017 [41], population density is calculated by the number of people per square km, denoted by Density.

Structure of population (Aging): Since the aging problem can change a government’s attitude to natural environmental protection, we measure the aging problem by the share of people aged 65 or above in relation to total population (Wang et al., 2021a) [42], denoted by Aging.

Industrial share (IND): To control the influence of industrial structure on carbon emissions, we measure industrial structures by the proportion of industry value added (including construction) to GDP (Usman et al., 2021) [43].

Process of urbanization (Urban): As suggested by Dale (2018) [44], we use the share of urban residents to total population to measure the urbanization rate, which can capture the potential influence of urbanization on carbon emissions.

International trade (Trade): Similarly to Li et al., (2017) [45], we utilize the share of exports and imports of goods and services as a proportion of GDP to capture the degree of openness, denoted by Trade.

Utilization of foreign investment (IFDI): To measure production activities created by foreign direct investment, we include the control variable of FDI as calculated by the ratio of net inward FDI to GDP, which is supported by Mahadevan and Sun (2020) [46].

Table 1 provides the details of such variables.

### 2.2. Data

Data on CO_2_ are sourced from the database of the World Bank. Data on Uncertainty are provided by Ahir et al., (2018) [34] (Ahir et al., (2018) [34], The world uncertainty index, Website of World Uncertainty Index: http://www.policyuncertainty.com/wui_quarterly.html, accessed on 13 November 2021). Data for GI is obtained from the OECD (2020) [48], while data for remaining variables are given by World Development Indicators (WDI (2020) [47] World Bank, World Development Indicators. http://databank.worldbank.org/data/reports.aspx?source=wdi-database-archives-(beta), accessed on 13 November 2021). We merge all data together according to dimensions such as country and year, and then filter the observations by following principles such as dropping observations with missing values. We finally construct a panel dataset for 137 countries from 1970 to 2018. Similar to Wang et al., (2021b) [38], we take the log for these variables.

The basic distribution of the variables is listed in Table 2. For the CO_2_ variable, it is obvious that the mean for CO_2_ is 3.133, and the median of is 3.071, while the minimum, standard deviation (S.D), and maximum are 0.047, 1.953, and 9.206, respectively, which means that CO_2_ varies greatly among different countries. When we analyze the distribution of EPU, we find that the min, mean, median and max of Uncertainty are 0, 0.127, 0.1 and 0.851, respectively, while S.D is 0.112.

### 2.3. Estimating Methods

According to the previous literature, such as Wen et al., (2016) [37] and Wang et al., (2021b) [38], carbon emissions are not only influenced by current situations, but are also affected by previous environmental performance; to include this dynamic process into our estimation, we choose the generalized moment method to conduct empirical investigations. However, as suggested by Wang et al., (2019) [49], the GMM estimation system has some advantages over other estimation methods, so we finally utilized the GMM estimation system to conduct our empirical investigation, which is given as following.

(1) $${{CO}_{2}}_{it}=\alpha_{1}{{CO}_{2}}_{i,t-1}+\theta Uncertainty_{it}+\beta^{\prime }X+u_{i}+u_{t}+\varepsilon_{it}$$

where i=1,2,3…N stand for the individual country, whereas t=1,2,3…T represent the dimension of year. ${CO}_{2}$ is the dependent variable, while ${{CO}_{2}}_{i,t-1}$ is the first lag. Uncertainty is the variable of EPU. *X* represents the control variables, β is the vector for the corresponding coefficients, and $u_{i}$ and $u_{t}$ stands for the fixed effect of individual country and year, respectively.

## 3. Results and Discussion

### 3.1. Baseline Results

At first, we estimate the standard STIRAT model with EPU by using the GMM estimation system, whose results are shown in column (1) of Table 3. The coefficient of uncertainty is 0.138, which is significantly positive even at 1%, indicating that the EPU would bring about more CO_2_ emissions. When we take other variables for the extended STIRPAT model into account, the coefficient of uncertainty in column (2)–(4) is 0.171, 0.182, 0.172, respectively, and all are significantly positive at 1%, again confirming the idea that EPU would cause more CO_2_ emissions.

These GMM estimation results strongly support that EPU exerts a positive impact on CO_2_ emissions, supporting previous literature such as that of Adams et al., (2020) [20] and Qi et al., (2021) [36]. The potential reason for this is that, once the EPU increases, the more the attention of policymakers would turn to how to maintain economic growth by stimulating economic activities, thus reducing the importance of environmental protection during the implementation of these policies. Furthermore, with increasing EPU, the activity of research and development in clean energy and energy-saving technologies experiences some decline and firms prefer to use cheap fossil fuels (Ulucak and Khan, 2020) [50], which may cause more carbon emissions (Al-Thaqeb and Algharabali, 2019) [51].

### 3.2. Robustness Test

To prove our idea that EPU positively affects CO_2_ emissions, we further carried out several robustness tests.

#### 3.2.1. Other Variables of EPU

Firstly, to avoid the potential bias caused by the measurement, we utilize another three variables to capture the EPU, provided by Ahir et al., (2018) [34]. The first is trade EPU, denoted by uncertainty_trade; the second is the absolute count of the word “uncertainty” in the quarterly Economist Intelligence Unit (EIU) country reports, denoted by uncertainty_absolute; while the third is similar to the EPU, but is adjusted by the nearest two seasons, denoted by uncertainty_season. (More detailed information on such variables can be seen in: http://www.policyuncertainty.com/media/WUI_mimeo_10_29.pdf, accessed on 13 November 2021) Table 4 provides the estimation results when we utilize these three variables to measure the EPU. From Table 4 we can obtain that the coefficient of uncertainty_trade is 0.059, which passes the significance test at 5%, suggesting that a higher trade EPU often brings more CO_2_ emissions. Moreover, the coefficient of uncertainty_absolute and uncertainty_season in column (2) and (3) is 0.023 and 0.083, respectively, offering strong evidence for the conclusion that EPU would lead to more CO_2_ emissions.

#### 3.2.2. Changing the Measurement of Carbon Emissions

Secondly, we re-conduct the empirical examination by utilizing another three measurements of GHG emissions, total GHG emissions, CH_4_ emissions and N_2_O emissions, as suggested by Lin et al., (2017) [52]. We offer the results in Table 5. While we control the time fixed effect, individual fixed effect and other variables which may affect CO_2_ emissions, the coefficient of uncertainty in column (1) is 0.293, which is significantly positive, again indicating that the EPU would bring about more CO_2_ emissions. Furthermore, while we capture the carbon emissions by CH_4_ and N_2_O, whose results are listed in column (2)–(3), we can obtain the coefficient of Uncertainty in column (2)–(3) as 0.103, 0.025, respectively, which passes the significance test at the 5% level, suggesting that EPU exerts a significantly positive influence on carbon emissions.

#### 3.2.3. Panel Fixed Effect (FE) Estimation

Thirdly, to avoid the potential bias caused by the estimation method, we re-estimated the model by employing panel FE estimation which incorporates the individual fixed effect and time fixed effect, similar to Chu and Le (2021) [53], who also utilized this estimation to examine the impact of EPU on carbon emissions. We offer the results of panel FE estimation in Table 6. While we control the time fixed effect, individual fixed effect and other variables which may affect the environmental performance, the coefficient of uncertainty in column (1) is 8.246, which is significantly positive, again indicating that the EPU would bring about more carbon emissions. Furthermore, while we capture the EPU by employing the other three variables, whose results are listed in column (2)–(4), we can obtain that the coefficient of uncertainty_trade, uncertainty_absolute, and uncertainty_season in columns (2)–(4) is 0.021, 0.003, and 0.004, respectively, which passes the significance test at the 5% level, also suggesting that EPU exerts a significantly positive effect on carbon emissions. In summary, the results of panel FE estimation are similar to those of GMM estimation in Table 3 and Table 4, confirming the reliability of our baseline finding.

#### 3.2.4. New Sample

Thirdly, to avoid the potential bias caused by outliers, we re-constructed new sub-samples by dropping observations with values smaller than 10% CO_2_ or larger than 10% CO_2_, according to Wang et al., (2021a) [42]. We hence re-estimate the GMM based on such a new sample and display the results in Table 7. It is easily to see that the coefficient of uncertainty, uncertainty_trade, uncertainty_absolute and uncertainty_season are negative and pass the significance test at the 10% level, indicating that the results based on the middle 80% sample also support the positive influence of EPU on carbon emissions.

## 4. Conclusions

To investigate the influence of EPU on CO_2_ emissions, we collected the cross-country data for 137 countries from 1970 to 2018 to carry out a GMM system estimation. The baseline result confirms that the EPU would bring about more CO_2_ emissions, which is also credible when we utilize the new measurement of EPU or the new measurement for CO_2_ emissions. In addition, while we examine the moderating effect of other factors in EPU’s influence on CO_2_ emissions, we see that the effect of EPU on air pollution among OECD countries is lower than that in non-OECD countries, suggesting that a higher level of economic development would reduce the environmental adverse effect of EPU. Besides, higher globalization and more international trade would weaken the effect of EPU on CO_2_ emissions, and better governance would reduce EPU’s influence on CO_2_ emissions. Aside from this, we also test whether the role of EPU in CO_2_ emissions varies among different political regimes; the results support that the influence of EPU on globalization is stronger in autocracies than that in democracies, whereas left-wing governments can somewhat reduce EPU’s influence on CO_2_ emissions.

According to our empirical findings, we offer the following policy implications to improve environmental performance. Firstly, given that EPU would have some negative influence on environmental performance, governments could put in more effort to protect the stability and predictability of economic policies, especially environmentally friendly policies; if they prefer to conduct new policies, the transition should be smooth, especially during election periods or when experiencing significant external shock such as coronavirus; the stability of economic policies can not only spur economic incentives, but also improve environmental performance, which are both essential for national sustainable development. Secondly, among the specific dimension of EPU, trade uncertainty also negatively affects environmental performance, and during the era of post coronavirus, trade uncertainty also increases. To reduce the negative effect of trade uncertainty, policymakers should continue their earlier preference for international trade or trade among domestic country, and conduct long-term policies to maintain sustainable environments for trade activities, which are beneficial for environmental protection.

Even if our study comprehensively investigated the impact of economic policy uncertainty on carbon emissions by using 137 countries and reported that EPU causes more carbon emissions, this paper did not uncover the transmission channels through which EPU can affect carbon emissions, nor whether the EPU effect on carbon emissions is constant among different countries, which need to be further investigated in future studies.

## Figures and Tables

**Table 1:** Variable definition.

Variables	Definition	Source
Dependent variables		
CO_2_	The total amount of CO_2_ emissions	WDI (2020) [47]
Independent Variables		
Uncertainty	The world uncertainty index	Ahir et al., (2018)
Control variables		
GDP	GDP per capita (constant 2010 US$)	WDI (2020) [47]
POP	The number of total population, whose unit is million	WDI (2020) [47]
GI	The number of patent applications about the environmental management	OECD (2020) [48]
Density	Population density (people per sq. km of land area)	WDI (2020) [47]
Aging	Population ages 65 and above (% of total population)	WDI (2020) [47]
IND	Industry (including construction), value added (% of GDP)	WDI (2020) [47]
Urban	Urban population (% of total population)	WDI (2020) [47]
Trade	Share of the sum of exports and imports of goods and services to GDP	WDI (2020) [47]
IFDI	Foreign direct investment, net inflows (% of GDP)	WDI (2020) [47]

**Table 2:** Summary of descriptive statistics.

Variable	N	Mean	Min	P25	Median	P75	Max	S. D
CO_2_	4814	3.133	0.047	1.463	3.071	4.569	9.206	1.953
Uncertainty	4814	0.127	0.000	0.044	0.100	0.185	0.851	0.112
GDP	4814	8.201	5.306	6.979	8.117	9.292	11.431	1.501
POP	4814	16.362	13.049	15.377	16.133	17.261	21.055	1.374
GI	4814	0.996	0.000	0.000	0.000	1.386	8.203	1.738
Density	4814	3.986	0.748	3.076	4.089	4.800	8.981	1.375
Aging	4814	1.891	0.522	1.438	1.687	2.417	3.353	0.573
IND	4814	3.309	0.835	3.110	3.316	3.518	4.486	0.383
Urban	4814	3.843	1.347	3.532	3.996	4.295	4.615	0.581
Trade	4814	4.146	0.155	3.836	4.153	4.504	6.095	0.575
IFDI	4814	1.088	0.000	0.449	1.002	1.573	4.648	0.785

**Table 3:** SYS-GMM estimations for EPU’s impact on EPI.

	(1)	(2)	(3)	(4)
L. CO_2_	0.754 ***	0.717 ***	0.505 ***	0.523 ***
	(23.278)	(17.718)	(9.612)	(9.848)
Uncertainty	0.138 ***	0.171 ***	0.182 ***	0.172 ***
	(4.008)	(4.208)	(3.688)	(3.586)
GDP	0.313 ***	0.430 ***	0.465 ***	0.496 ***
	(6.034)	(6.617)	(5.533)	(5.746)
POP	0.522 ***	−1.657 ***	−7.231 ***	−6.725 ***
	(6.768)	(−3.210)	(−6.855)	(−6.604)
GI	−0.007 ***	−0.006 ***	−0.005 ***	−0.004 *
	(−3.168)	(−3.091)	(−2.603)	(−1.949)
Density		1.975 ***	7.262 ***	6.771 ***
		(3.848)	(7.103)	(6.752)
Aging		−0.730 ***	−1.628 ***	−1.513 ***
		(−3.858)	(−5.329)	(−5.231)
IND			0.008	0.007
			(0.625)	(0.432)
Urban			0.354 ***	0.276 ***
			(5.945)	(4.464)
Trade				−0.004
				(−0.260)
IFDI				−0.001
				(−0.241)
Year FE	Yes	yes	yes	yes
N	4667	4667	4667	4667
AR (1)	−4.787	−4.749	−3.911	−4.023
AR (1)-P	0.000	0.000	0.000	0.000
AR (2)	−1.346	−1.283	−0.926	−0.979
AR (2)-P	0.178	0.200	0.354	0.327
Hansen-P	0.954	0.946	0.983	0.974

Notes: *** and * indicate statistical significance at the 1%, and 10% levels, respectively. Z-statistics are in parenthesis.

**Table 4:** Robustness test—changing the measurement of EPU.

	(1)	(2)	(3)
L. CO_2_	0.403 ***	0.506 ***	0.518 ***
	(3.313)	(8.662)	(9.758)
Uncertainty_trade	0.059 **		
	(2.244)		
Uncertainty_absolute		0.023 ***	
		(3.390)	
Uncertainty_season			0.083 *
			(1.667)
GDP	0.983 ***	0.451 ***	0.514 ***
	(5.543)	(5.259)	(5.878)
POP	10.477 *	−6.987 ***	−6.327 ***
	(1.663)	(−5.943)	(−6.312)
GI	0.002	−0.005 **	−0.004 *
	(0.608)	(−2.239)	(−1.839)
Density	−9.044	7.001 ***	6.341 ***
	(−1.457)	(6.162)	(6.430)
Aging	−0.453 *	−1.438 ***	−1.541 ***
	(−1.658)	(−4.428)	(−5.263)
IND	−0.022	0.008	0.005
	(−0.792)	(0.483)	(0.316)
Urban	−0.095	0.375 ***	0.282 ***
	(−0.460)	(4.881)	(4.541)
Trade	0.007	0.004	−0.003
	(0.537)	(0.265)	(−0.208)
IFDI	−0.005	−0.002	−0.001
	(−1.198)	(−0.457)	(−0.181)
Year FE	yes	yes	yes
N	2818	4670	4667
AR (1)	−2.964	−4.095	−3.908
AR (1)-P	0.003	0.000	0.000
AR (2)	−0.169	−0.922	−0.920
AR (2)-P	0.866	0.356	0.357
Hansen-P	0.733	0.926	0.960

Notes: ***, ** and * indicate statistical significance at the 1%, 5%, and 10% levels, respectively. Z-statistics are in parenthesis.

**Table 5:** Robustness test—changing the measurement of dependent variables.

	(1) GHG	(2) CH_4_	(3) N_2_O
L.dependent	0.440 ***	0.416 ***	0.345 ***
	(9.354)	(11.024)	(6.126)
Uncertainty	0.293 **	0.103 **	0.025 **
	(1.978)	(2.573)	(2.456)
GDP	0.375 ***	0.070 **	0.042
	(4.960)	(1.973)	(0.570)
POP	8.176 ***	1.307	5.663 **
	(3.469)	(1.269)	(2.249)
GI	−0.002	−0.001	−0.003
	(−0.301)	(−0.909)	(−1.251)
Density	−7.379 ***	−0.678	−5.199 **
	(−3.038)	(−0.649)	(−2.092)
Aging	0.200	0.189 **	0.106
	(0.781)	(2.112)	(0.363)
IND	0.053 *	0.000	0.011
	(1.785)	(0.019)	(0.712)
Urban	−0.379 ***	0.049	0.134 **
	(−5.771)	(1.333)	(2.530)
Trade	0.013	0.007	0.030 ***
	(0.670)	(1.157)	(3.281)
IFDI	−0.002	−0.001	−0.003
	(−0.622)	(−0.687)	(−1.202)
Year FE	yes	yes	yes
N	3021	3276	3276
AR (1)	−2.643	−2.664	−3.275
AR (1)-P	0.008	0.008	0.001
AR (2)	1.231	2.418	1.630
AR (2)-P	0.218	0.016	0.103
Hansen-P	0.403	0.388	0.323

Notes: ***, ** and * indicate statistical significance at the 1%, 5%, and 10% levels, respectively. T-statistics are in parenthesis.

**Table 6:** Robustness test—changing the estimation.

	(1)	(2)	(3)	(4)
Uncertainty	8.246 ***			
	(6.003)			
Uncertainty_trade		0.021 **		
		(2.437)		
Uncertainty_absolute			0.003 **	
			(2.320)	
Uncertainty_season				0.004 ***
				(3.081)
GDP	0.636 ***	0.618 ***	0.636 ***	0.636 ***
	(6.743)	(6.601)	(6.724)	(6.748)
POP	0.727	2.005	0.726	0.728
	(0.936)	(1.648)	(0.932)	(0.936)
GI	0.023	0.031 **	0.023	0.023
	(1.292)	(2.597)	(1.293)	(1.290)
Density	0.904	−0.676	0.906	0.904
	(1.232)	(−0.555)	(1.232)	(1.232)
Aging	0.686 ***	0.297 *	0.686 ***	0.686 ***
	(4.390)	(1.820)	(4.392)	(4.391)
IND	0.072	0.093 **	0.071	0.072
	(1.089)	(2.101)	(1.086)	(1.091)
Urban	0.132	1.029 ***	0.133	0.132
	(0.670)	(5.492)	(0.675)	(0.669)
Trade	0.082	0.008	0.082 *	0.082
	(1.653)	(0.317)	(1.663)	(1.652)
IFDI	−0.010	0.018	−0.010	−0.010
	(−0.873)	(1.628)	(−0.867)	(−0.872)
Year FE	yes	yes	yes	yes
Country FE	yes	yes	yes	yes
Cons	−19.363 **	−36.814 **	−19.341 **	−19.371 **
	(−2.004)	(−2.457)	(−1.998)	(−2.005)
N	4814	2956	4814	4814
R2	0.789	0.684	0.789	0.789
F	19.50	21.38	19.57	19.35

Notes: ***, ** and * indicate statistical significance at the 1%, 5%, and 10% levels, respectively. T-statistics are in parenthesis.

**Table 7:** Robustness test—middle 80% sample.

	(1)	(2)	(3)	(4)
L.CO_2_	0.433 ***	0.608 ***	0.522 ***	0.524 ***
	(7.721)	(24.505)	(14.645)	(14.766)
Uncertainty	0.026 ***			
	(3.556)			
Uncertainty_trade		0.052 ***		
		(5.262)		
Uncertainty_absolute			0.005 ***	
			(2.979)	
Uncertainty_season				0.086 *
				(1.840)
GDP	0.743 ***	0.329 ***	0.236 ***	0.233 ***
	(7.941)	(12.833)	(5.075)	(4.960)
POP	−6.951 ***	−0.618	−5.807 ***	−6.473 ***
	(−4.812)	(−0.652)	(−3.893)	(−3.639)
GI	−0.006 **	0.001	−0.005	−0.001
	(−2.430)	(0.138)	(−0.414)	(−0.077)
Density	7.644 ***	1.742 *	6.260 ***	6.915 ***
	(5.134)	(1.856)	(4.685)	(4.311)
Aging	−0.744 ***	0.288 ***	−0.455 **	−0.487 **
	(−2.586)	(3.519)	(−2.347)	(−2.398)
IND	−0.032	0.055 **	−0.011	−0.016
	(−1.617)	(2.399)	(−0.225)	(−0.312)
Urban	0.596 ***	0.278 ***	0.733 **	0.783 **
	(4.498)	(3.382)	(2.295)	(2.141)
Trade	0.007	−0.004	0.008	0.007
	(0.429)	(−0.810)	(1.081)	(0.990)
IFDI	−0.002	0.005	0.012 *	0.012 *
	(−0.404)	(1.507)	(1.775)	(1.731)
Year FE	yes	yes	yes	yes
N	3556	2248	3556	3556
AR (1)	−4.540	−5.508	−5.610	−5.627
AR (1)-P	0.000	0.000	0.000	0.000
AR (2)	−0.716	−0.900	−0.822	−0.761
AR (2)-P	0.474	0.368	0.411	0.447
Hansen-P	0.983	0.674	1.000	1.000

Notes: ***, **, and * indicate statistical significance at the 1%, 5%, and 10% levels, respectively. Z-statistics are in parenthesis.

## Data Availability

The data used to support the findings of this study are available from the corresponding author upon request.

## References

[1] Jordà Ò., Singh S.R., Taylor A.M. (2020). Longer-run economic consequences of pandemics?. Rev. Econ. Stat..

[2] Bakas D., Triantafyllou A. (2020). Commodity price volatility and the economic uncertainty of pandemics. Econ. Lett..

[3] Baker S.R., Farrokhnia R.A., Meyer S., Pagel M., Yannelis C. (2020). How does household spending respond to an epidemic? Consumption during the 2020 COVID-19 pandemic. Rev. Asset Pricing Stud..

[4] Balcilar M., Gupta R., Segnon M. (2016). The role of economic policy uncertainty in predicting US recessions: A mixed-frequency Markov-switching vector autoregressive approach. Economics.

[5] Degiannakis S., Filis G., Panagiotakopoulou S. (2018). Oil price shocks and uncertainty: How stable is their relationship over time?. Econ. Model..

[6] Hailemariam A., Smyth R., Zhang X. (2019). Oil prices and economic policy uncertainty: Evidence from a nonparametric panel data model. Energy Econ..

[7] Phan D.H.B., Iyke B.N., Sharma S.S., Affandi Y. (2021). Economic policy uncertainty and financial stability–Is there a relation?. Econ. Model..

[8] Guidolin M., La Ferrara E. (2010). The economic effects of violent conflict: Evidence from asset market reactions. J. Peace Res..

[9] Kang W., Ratti R.A., Vespignani J.L. (2017). Oil price shocks and policy uncertainty: New evidence on the effects of US and non-US oil production. Energy Econ..

[10] Olanipekun I., Olasehinde-Williams G., Saint Akadiri S. (2019). Gasoline prices and economic policy uncertainty: What causes what, and why does it matter? Evidence from 18 selected countries. Environ. Sci. Pollut. Res..

[11] Akron S., Demir E., Díez-Esteban J.M., García-Gómez C.D. (2020). Economic policy uncertainty and corporate investment: Evidence from the US hospitality industry. Tour. Manag..

[12] Salahuddin M., Alam K., Ozturk I., Sohag K. (2018). The effects of electricity consumption, economic growth, financial development and foreign direct investment on CO_2_ emissions in Kuwait. Renew. Sustain. Energy Rev..

[13] Shahbaz M., Mahalik M.K., Shahzad S.J.H., Hammoudeh S. (2019). Does the environmental K uznets curve exist between globalization and energy consumption? G lobal evidence from the cross-correlation method. Int. J. Financ. Econ..

[14] Kompas T., Pham V.H., Che T.N. (2018). The effects of climate change on GDP by country and the global economic gains from complying with the Paris climate accord. Earth’s Future.

[15] Coskuner C., Paskeh M.K., Olasehinde-Williams G., Akadiri S.S. (2020). Economic and social determinants of carbon emissions: Evidence from organization of petroleum exporting countries. J. Public Aff..

[16] Hambira W.L., Saarinen J., Moses O. (2020). Climate change policy in a world of uncertainty: Changing environment, knowledge, and tourism in Botswana. Afr. Geogr. Rev..

[17] Huo T., Li X., Cai W., Zuo J., Jia F., Wei H. (2020). Exploring the impact of urbanization on urban building carbon emissions in China: Evidence from a provincial panel data model. Sustain. Cities Soc..

[18] Jiang Y., Zhou Z., Liu C. (2019). Does economic policy uncertainty matter for carbon emission? Evidence from US sector level data. Environ. Sci. Pollut. Res..

[19] Yu J., Shi X., Guo D., Yang L. (2021). Economic policy uncertainty (EPU) and firm carbon emissions: Evidence using a China provincial EPU index. Energy Econ..

[20] Adams S., Adedoyin F., Olaniran E., Bekun F.V. (2020). Energy consumption, economic policy uncertainty and carbon emissions; causality evidence from resource rich economies. Econ. Anal. Policy.

[21] Gill A.R., Hassan S., Viswanathan K.K. (2019). Is democracy enough to get early turn of the environmental Kuznets curve in ASEAN countries?. Energy Environ..

[22] Shabir M., Ali M., Hashmi S.H., Bakhsh S. (2021). Heterogeneous effects of economic policy uncertainty and foreign direct investment on environmental quality: Cross-country evidence. Environ. Sci. Pollut. Res..

[23] Adedoyin F.F., Zakari A. (2020). Energy consumption, economic expansion, and CO_2_ emission in the UK: The role of economic policy uncertainty. Sci. Total Environ..

[24] Chen X., Zhang S., Ruan S. (2021). Polycentric structure and carbon dioxide emissions: Empirical analysis from provincial data in China. J. Clean. Prod..

[25] Doğan E., Güler C. (2021). How Economic Policy Uncertainty Affect Carbon Emissions: A Case of G-7 Countries. J. Econ. Bus. Issues.

[26] Abbasi K.R., Adedoyin F.F. (2021). Do energy use and economic policy uncertainty affect CO_2_ emissions in China? Empirical evidence from the dynamic ARDL simulation approach. Environ. Sci. Pollut. Res..

[27] Liu Y., Zhang Z. (2021). How does economic policy uncertainty affect CO_2_ emissions? A regional analysis in China. Environ. Sci. Pollut. Res..

[28] Baker S.R., Bloom N., Davis S.J. (2016). Measuring economic policy uncertainty. Q. J. Econ..

[29] Atsu F., Adams S. (2021). Energy consumption, finance, and climate change: Does policy uncertainty matter?. Econ. Anal. Policy.

[30] Pirgaip B., Dinçergök B. (2020). Economic policy uncertainty, energy consumption and carbon emissions in G7 countries: Evidence from a panel Granger causality analysis. Environ. Sci. Pollut. Res..

[31] Sohail M.T., Xiuyuan Y., Usman A., Majeed M.T., Ullah S. (2021). Renewable energy and non-renewable energy consumption: Assessing the asymmetric role of monetary policy uncertainty in energy consumption. Environ. Sci. Pollut. Res..

[32] Amin A., Dogan E. (2021). The role of economic policy uncertainty in the energy-environment nexus for China: Evidence from the novel dynamic simulations method. J. Environ. Manag..

[33] Anser M.K., Apergis N., Syed Q.R. (2021). Impact of economic policy uncertainty on CO_2_ emissions: Evidence from top ten carbon emitter countries. Environ. Sci. Pollut. Res..

[34] Ahir H., Bloom N., Furceri D. (2018). The World Uncertainty Index. Available at SSRN 3275033. https://papers.ssrn.com/sol3/papers.cfm?abstract_id=3275033.

[35] Shao S., Yang L., Yu M., Yu M. (2011). Estimation, characteristics, and determinants of energy-related industrial CO_2_ emissions in Shanghai (China), 1994–2009. Energy Policy.

[36] Qi X., Wang X., Jin X., Wang Z.M., Zhang B., Wen C. (2021). Will Policy Uncertainty Deteriorate Haze Pollution? A Spatial Spillover Perspective. Int. J. Environ. Res. Public Health.

[37] Wen J., Hao Y., Feng G.-F., Chang C.-P. (2016). Does government ideology influence environmental performance? Evidence based on a new dataset. Econ. Syst..

[38] Wang Q.-J., Geng Y., Xia X.-Q. (2021). Revisited Globalization’s Impact on Total Environment: Evidence Based on Overall Environmental Performance Index. Int. J. Environ. Res. Public Health.

[39] Yang Q.-C., Feng G.-F., Chang C.-P., Wang Q.-J. (2021). Environmental protection and performance: A bi-directional assessment. Sci. Total Environ..

[40] Verma A., Schmidt-Vogt D., De Alban J.D.T., Lim C.L., Webb E.L. (2021). Drivers and mechanisms of forest change in the Himalayas. Glob. Environ. Chang..

[41] Bottero A., D’Amato A.W., Palik B.J., Bradford J.B., Fraver S., Battaglia M.A., Asherin L.A. (2017). Density-dependent vulnerability of forest ecosystems to drought. J. Appl. Ecol..

[42] Wang Q.-J., Feng G.-F., Wang H.-J., Chang C.-P. (2021). The impacts of democracy on innovation: Revisited evidence. Technovation.

[43] Usman M., Anwar S., Yaseen M.R., Makhdum M.S.A., Kousar R., Jahanger A. (2021). Unveiling the dynamic relationship between agriculture value addition, energy utilization, tourism and environmental degradation in South Asia. J. Public Aff..

[44] Dale S. (2018). Urban bird community composition influenced by size of urban green spaces, presence of native forest, and urbanization. Urban Ecosyst..

[45] Li L., Liu J., Long H., de Jong W., Youn Y.-C. (2017). Economic globalization, trade and forest transition-the case of nine Asian countries. For. Policy Econ..

[46] Mahadevan R., Sun Y. (2020). Effects of foreign direct investment on carbon emissions: Evidence from China and its Belt and Road countries. J. Environ. Manag..

[47] WDI (2020). World Development Indicators.

[48] OECD (2020). OECD Statistics.

[49] Wang Q.-J., Feng G.-F., Chen Y.E., Wen J., Chang C.-P. (2019). The impacts of government ideology on innovation: What are the main implications?. Res. Policy.

[50] Ulucak R., Khan S.U.D. (2020). Relationship between energy intensity and CO_2_ emissions: Does economic policy matter?. Sustain. Dev..

[51] Al-Thaqeb S.A., Algharabali B.G. (2019). Economic policy uncertainty: A literature review. J. Econ. Asymmetries.

[52] Lin S., Sun J., Marinova D., Zhao D. (2017). Effects of population and land urbanization on China’s environmental impact: Empirical analysis based on the extended STIRPAT model. Sustainability.

[53] Chu L.K., Le N.T.M. (2021). Environmental quality and the role of economic policy uncertainty, economic complexity, renewable energy, and energy intensity: The case of G7 countries. Environ. Sci. Pollut. Res..

